# Smoothed Cepstral Peak Prominence: A Comparison Between Dysphonic and Non-dysphonic Mexican Adults Employing the Praat Software

**DOI:** 10.7759/cureus.72292

**Published:** 2024-10-24

**Authors:** José Rosmal Cortés Ponce, Luis Ángel Garza Montelongo, Jorge Eduardo Juárez Silva, José Luis Trevino González

**Affiliations:** 1 Otolaryngology-Head and Neck Surgery Universitary Center, Hospital Universitario "Dr. José Eleuterio González", Monterrey, MEX

**Keywords:** cepstral peak prominence, dysphonia, laryngeal pathology, speech, vocal assessment

## Abstract

Background

Acoustic vocal analysis provides objective and measurable values for various voice parameters, such as fundamental frequency (F0), shimmer, jitter, and the noise-to-harmony ratio (NHR). In severely dysphonic patients, who present increased variability in glottic cycles and abnormalities in vocal intensity, conventional acoustic analysis is an unreliable predictor of dysphonia. The logarithmic transformation of the vocal spectrum (cepstrum) allows capturing the signal without relying on recording technique, frequency, or vocal intensity. Smoothed cepstral peak prominence (CPPS) is a variable in cepstral analysis that serves as a reliable predictor of dysphonia, being directly proportional to its severity.

Objective

To determine the mean value of CPPS in continuous speech and sustained vowels in dysphonic and non-dysphonic Mexican adults, with or without laryngeal pathology.

Materials and methods

Sustained vowel and continuous speech analysis was performed using the Praat software (Version 6.1.15, developed by Paul Boersma and David Weenink, Phonetic Sciences, University of Ámsterdam, https://www.praat.org/) to obtain the smoothed cepstral peak prominence (CPPS) in 51 dysphonic patients with laryngeal pathology and 24 non-dysphonic patients without underlying laryngeal pathology. Frequency perturbation variables such as shimmer, jitter, and NHR were collected. Quality of life was assessed using the 30-item Voice Handicap Index (VHI-30).

Results

We found significantly lower median CPPS values in patients with laryngeal pathology, both in sustained vowel (6.59, IQR 4.09-9.38, p < 0.001) and in connected speech (4.82, IQR 3.57-6.03, p < 0.001), compared with the non-dysphonic population (sustained phonation: 11.69, IQR 9.26-12.81, p < 0.001; connected speech: 6.38, IQR 0.90-7.09, p < 0.001).

Conclusions

A low CPPS value is a reliable predictor of underlying laryngeal pathology in dysphonic voices and could be considered for routine screening and diagnosis in patients with vocal pathology.

## Introduction

The prevalence of vocal disorders is as high as 50% in the general population [1]. The clinical assessment of patients with dysphonia involves a multidimensional approach that includes a detailed anamnesis, an auditory-perceptual analysis, a quantitative acoustic analysis, a qualitative voice signal assessment, self-evaluation of voice using validated instruments, and a structural and functional visual laryngeal examination [2].

The auditory-perceptual analysis is considered the gold standard method for determining the presence or absence of dysphonia [3]. Unfortunately, the results depend on the experience, familiarity, auditory interpretation, and scale used by the evaluator, which can lead to inter-rater variability and, therefore, misdiagnosis [4].

The acoustic vocal analysis assessment has the advantage of being a non-invasive, reproducible, low-cost, and objective method. This analysis utilizes two types of voice sampling: the phonation of a sustained vowel and connected speech, which is the spoken language used in a continuous sequence. The sampling of connected speech has the advantage of evaluating different voice and sound styles that are more representative of the patient’s voice. In contrast, sustained vowel measures a more controlled and stable but less natural phonation [5].

The main unit of the vocal signal in acoustic analysis is the sine wave, which has frequency (number of cycles per second, correlated with pitch) and amplitude (magnitude of the wave deflection, correlated with intensity). If the amplitude of the waves is plotted as a function of frequency, a vocal spectrum is produced. A constant and unchanging frequency signal is called periodic, but if the frequency inconsistently varies with time, the signal is said to be aperiodic [6].

The most common measures of voice quality are time-based parameters such as jitter (frequency perturbation), shimmer (amplitude perturbation), and noise-to-harmony ratio (mean energy relation of the harmonic components versus the non-harmonic). Unfortunately, these parameters are low-reliable predictors of dysphonia, as they require precise identification of the cycles in frequency for their analysis, making them non-applicable to severely dysphonic voices with increased aperiodicity. Additionally, the reliability of these parameters when applied to connected speech is compromised due to the presence of muted phonemes, changes in voice articulation, and variations in prosody and tone [1,6-8]. As a result, it is possible that acoustic analysis using only sustained vocalizations does not accurately assess real voice quality.

To overcome the limitations of conventional acoustic analysis, frequency-independent acoustic measures have been developed; among them, the most notable are those derived from the cepstrum [4,9]. This is the inverse transformation of the logarithm of the signal spectrum's power. The cepstral analysis serves as an acoustic indicator for quantifying the fundamental frequency based on the harmonic sound components. In the cepstrum, the 'frequency' of each waveform component of the spectrum is referred to as 'quefrency.' These uniformly spaced frequencies with lower amplitude peaks in the cepstrum are termed harmonics. The peak with the highest amplitude is known as the cepstral peak. When a linear regression line representing the average sound energy is plotted across the cepstrum, the distance from the cepstral peak to this linear regression line is referred to as the cepstral peak prominence (CPP), measured in decibels [5,10]. The smoothed cepstral peak prominence (CPPS) is a modification of the CPP, which involves averaging the cepstral magnitude across frequencies and time, and it is strongly correlated with the overall severity of dysphonia [1,3].

Introduced by Hillenbrand and Hound, the CPP has proven to be a reliable and valid parameter for assessing overall voice quality [11]. Many studies have shown that its use can differentiate dysphonic from non-dysphonic patients with high sensitivity and specificity, with ranges of 77-92% and 77-89%, respectively [3,6,11]; the Praat software (Version 6.1.15, 2018, developed by Paul Boersma and David Weenink, Phonetic Sciences, University of Ámsterdam, https://www.praat.org/), the Analysis of Dysphonia in Speech and Voice (ADSV) system (PENTAX Medical, Tokyo, Japan), and the Hillenbrand & Hound algorithm are the primary software applications used to obtain this parameter [12]. The usefulness of cepstral analysis is based on the fact that the more periodic the voice is, the more defined the harmonic configuration in the spectrum becomes, and therefore, the more prominent the cepstral peak. As a result, higher values of CPP and CPPS are associated with voices of better quality (more periodic voices), while lower values of CPP and CPPS are associated with voices of poorer quality (more aperiodic voices) [13-15].

## Materials and methods

A prospective, descriptive, comparative, and analytic study was performed in the Otolaryngology-Head and Neck Surgery Division of the University Hospital “Dr. José Eleuterio González," a tertiary care referral center in northeast Mexico. This study was assessed and approved by the Research and Institutional Ethics Committee of University Hospital “Dr. José Eleuterio González” with approval register OT24-0003.

Study population

This study included 51 Mexican patients older than 18 years old, recruited from March 2021 to June 2024, who attended the Otolaryngology-Head and Neck Surgery Division of the University Hospital “Dr. José Eleuterio González” and whose main complaint was dysphonia due to laryngeal pathology. Additionally, 24 patients without vocal abnormalities were recruited as a healthy control group. Exclusion criteria included patients under 18 years old, those diagnosed with rhinosinusitis, nasal polyposis, head and neck tumors, craniofacial malformations, tonsil hypertrophy, velopharyngeal insufficiency, or any extrinsic pathology that caused vocal abnormalities; tracheostomy users and patients who underwent laryngectomy were also excluded from this study.

Surveys

A self-vocal perception survey was applied, using the Spanish-validated version of the 30-item Voice Handicap Index (VHI-30), to report the voice disorder’s life quality impact, on its functional, physical, and emotional aspects [10].

Acoustic voice analysis

After providing a detailed explanation of the procedure and obtaining verbal informed consent, an acoustic voice analysis was conducted in a soundproof room with the patient seated. The recording was performed using a professional condenser microphone (MIC-650, Steren Inc., Mexico), positioned 10 cm from the mouth, and connected directly to a computer. Praat software version 6.2.09 (2021, Boersma P, Weenink D) was used for the vocal analysis.

We asked the patient to perform a sustained phonation of the vowel/a/ for six seconds; subsequently, the continuous speech was evaluated through the vocalization of four random phrases taken from the validated “Spanish Matrix Sentence Test" [16].

CPPS values were obtained. For its calculation, we employed the following configuration: Sound [vowels or phrases] analyze periodicity → PowerCepstrogram [Pitch floor = 60 Hz; time step = 0.002 s; maximum frequency = 5 000 Hz; pre-emphasis = 50 Hz] → windows = 0.01 s; quefrency averaging window = 0.001 s; peak search pitch range = 60 - 330 Hz; tolerance (0 - 1) = 0.05; interpolation = parabolic; tilt line quefrency range = 0.001 - 0.0 s; line type = straight; fit method = robust.

Frequency parameters were also collected: fundamental frequency, vocal intensity, shimmer, jitter, and noise-to-harmony ratio on continuous speech and sustained vowels. To calculate these parameters, we employed the following configuration: Sound [vowel or phrases] → View & Edit → Show Pitch - Intensity → Pulse → Voice Report.

All sociodemographic variables and voice analysis results were emptied in an Excel (Microsoft Office v. 2019; Microsoft Inc., Redmond, Washington, United States) database, using all variables in numeric formatting.

Statistical analysis

The descriptive statistic was reported on frequencies and percentages for categorical variables. For quantitative variables, central tendency measures and dispersion were applied to (mean/median, standard deviation/interquartile range). The distribution of the data was assessed using the Kolmogórov-Smirnov or Shapiro-Wilk tests. Categorical variables were evaluated using the chi-square test or Fisher’s exact test, while in quantitative variables the data was assessed applying the T-student or Wilcoxon rank-sum test. A binary logistic regression was applied to the results. Pearson or Spearman correlations were performed based on the distribution of the data. A p-value ≤ 0.05 and a 95% confidence interval (CI) were considered statistically significant. All statistical analyses were performed on IBM SPSS Statistics for Windows, Version 25 (Released 2017; IBM Corp., Armonk, New York, United States).

## Results

A total of 75 patients, ages 22-92, were evaluated. Fifty-one patients presented laryngeal pathology (males n = 24, 47.1%, females n = 27, 52.9%), and the rest of the 24 patients were included in the control group (males n = 13, 54.2%, females n = 11, 45.8%). The mean age in the study group was 53 years old (IQR 40-64) and 27 years in the control group (IQR 24-33.5). The underlying diagnoses of the studied population are presented in Table 1.

**Table 1:** Underlying laryngeal diagnosis. Results are presented as n (%).

Diagnosis	n	%
Laryngopharyngeal reflux	10	13.33%
Laryngeal carcinoma	9	12%
Unilateral vocal cord paralysis	10	13.33%
Bilateral vocal cord paralysis	2	2.66%
Tensional dysphonia	4	5.33%
Vocal cord polyp	5	6.66%
Vocal cord nodule	5	6.66%
Vocal cord papilloma	3	4%
Laryngeal tuberculosis	2	2.66%
Reinke edema	1	1.33%
Healthy control (No laryngeal diagnosis)	24	32%

A significantly increased mean of VHI-30 score was registered in the study group (59 points, p < 0.001) in comparison to the control group (1 point, p < 0.001).

In the acoustic analysis of the sustained vowel/a/, significantly lower CPPS scores were obtained in the study group compared to the control group (6.59 vs. 11.69, p < 0.001). Additionally, statistically significant differences were reported in the shimmer value (15.9% vs. 7.6%, p < 0.001), jitter value (92.71 μs vs. 19.8 μs, p < 0.001), NHR (0.2 vs. 0.05, p < 0.001), voice breaks (3.6% vs. 0%, p < 0.001), and vocal intensity (60.9 dB vs. 57.3 dB, p = 0.012) between the study and control groups, respectively. No differences were found in the fundamental frequency (152.2 Hz vs. 148.2 Hz, p = 0.95) between the two groups. Full sustained vowel analysis results are displayed in Table 2.

**Table 2:** Median and interquartile ranges (IQR) on sustained vowel analysis between dysphonic and control subjects. The results are presented as median (IQR). P-value reflects the results of Wilcoxon rank-sum analysis^a^. Bold significance p-value < 0.05. NS: not significant; jitter rap: relative average perturbation; jitter ppq5: five-point period perturbation quotient; jitter ddp: difference of differences of periods; shimmer apq3, apq5, and apq11: amplitude perturbation quotient 3, 5 and 11, respectively; shimmer dda: difference of differences amplitude; CPPS: smoothed cepstral peak prominence

Variable	Pathologic voice (n=51)	Non-pathologic voice (n=24)	W^a^	p
CPPS dB	6.59 (4.09-9.38)	11.69 (9.26-12.81)	1490	< 0.001
Intensity dB	60.9 (58.5-64)	57.3 (55-62.5)	691.5	0.012
Fundamental frequency Hz	152.2 (126.8-199.5)	148.2 (112.6-163.2)	NS	0.95
Tone (median) Hz	154.3 (126.9-204.2)	139.5 (103.4-171.1)	NS	0.063
Tone (mean) Hz	148.1 (126.8-199.1)	148.1 (112.6-163.2)	NS	0.126
Tone (SD) Hz	18.77 (4.59 - 47.48)	1.39 (0.77 - 18.94)	501	< 0.001
Tone (minimum value) Hz	96.95 (78.85 - 118.95)	110.59 (87.49 - 145.99)	NS	0.08
Tone (maximum value) Hz	213.8 (162.9-326.1)	151.5 (116.6-208.9)	592	< 0.001
Fraction of unvoiced frames %	6.1 (2.3-29.2)	0 (0-0)	397.5	< 0.001
Voice breaks %	3.6 (0.2-21.1)	0 (0-0.2)	560	< 0.001
Jitter (local) %	1.2 (0.6-3.2)	0.2 (0.2-0.6)	475	< 0.001
Jitter (local absolute) μs	92.71 (36.9 - 169.83)	19.98(15.75 - 60.31)	498	< 0.001
Jitter (rap) %	0.6 (0.3-1.8)	0.1 (0.1-0.3)	489	< 0.001
Jitter (ppq5) %	0.7 (0.3-1.8)	0.1 (0.1-0.3)	491.5	< 0.001
Jitter (ddp) %	2.0 (0.9-5.5)	0.4 (0.3-1.0)	497	< 0.001
Shimmer (local) %	15.9 (11.8-18.2)	7.6 (6.5-13.1)	549	< 0.001
Shimmer (local, dB)	1.4 (1.1-1.6)	0.6 (0.6-1.1)	540	< 0.001
Shimmer (apq3) %	8.1 (5.9-9.4)	4.0 (3.4-7.0)	562	< 0.001
Shimmer (apq5) %	9.6 (7.1-11.4)	4.8 (3.8-8.4)	574	< 0.001
Shimmer (apq11) %	11.8(9.9-13.9)	.6 (4.9-9.2)	548	< 0.001
Shimmer (dda) %	23.8 (17.6-28.2)	12.0 (10.3-21.1)	1575	< 0.001
Autocorrelation (mean)	0.8 (0.7-0.9)	0.9 (0.8-0.9)	1575	< 0.001
Noise-to-harmonics ratio dB	0.2 (0.1-0.4)	0.05 (0.02-0.1)	531	< 0.001
Harmonics-to-noise ratio dB	7.58 (4.51 - 11.34)	13.67 (9.6 - 16.6)	1330	< 0.001

In the evaluation of connected speech during acoustic analysis, significantly lower CPPS values were obtained in the study group compared to the control group (4.82 vs. 6.38, p < 0.001). Additionally, statistically significant differences were found in shimmer values (18% vs. 16.9%, p = 0.008), jitter values (204.67 μs vs. 134.99 μs, p = 0.003), and vocal intensity (61.9 dB vs. 58.6 dB, p < 0.001) between the study and control groups. No differences were found in fundamental frequency (179.1 Hz vs. 152.5 Hz, p = 0.07), noise-to-harmony ratio (NHR) (0.3 vs. 0.2, p = 0.052), or voice breaks (10 vs. 7.5, p = 0.058) between both groups (Table 3).

**Table 3:** Median and interquartile ranges (IQR) on continuous speech analysis between dysphonic and control subjects. The results are presented as median (IQR). P-value reflects the results of Wilcoxon rank-sum analysis^a^. Bold significance p-value < 0.05. NS: not significant; jitter rap: relative average perturbation; jitter ppq5: five-point period perturbation quotient; jitter ddp: difference of differences of periods; shimmer apq3, apq5, and apq11: amplitude perturbation quotient 3, 5 and 11, respectively; shimmer dda: difference of differences amplitude; CPPS: smoothed cepstral peak prominence

Variable	Pathologic voice (n=51)	Non-pathologic voice (n=24)	W^a^	p
CPPS dB	4.82 (3.57-6.03)	6.38 (0.90-7.09)	1430.5	< 0.001
Intensity dB	61.9 (59.1-64.9)	58.6 (55.2-62.0)	623.5	0.001
Fundamental frequency Hz	179.1 (147.6-212.6)	152.5 (126.4-194.2)	NS	0.07
Tone (median) Hz	184.53 (136.9 - 213.88)	153.5 (125.59 - 190.26)	NS	0.107
Tone (mean) Hz	173.1 (146.4-211.1)	152.5 (126.4-194.2)	NS	0.109
Tone (SD) Hz	33.5 (19.2-50.8)	18.4 (8.3-26.4)	602	0.001
Tone (minimum value) Hz	90.0 (80.1-101.1)	88.8 (82.8-112.9)	NS	0.541
Tone (maximum value) Hz	289.8 (240.9-355.9)	221.7 (156.1-279.1)	637	0.002
Fraction of voiceless frames %	23.6 (18.6-30.6)	21.6 (17.1-25.3)	NS	0.162
Voice breaks %	24.9 (15.6-36.3)	20.2 (15.6-29.6)	NS	0.271
Jitter (local) %	3.2 (2.6-4.3)	2.3 (1.7-2.5)	457	< 0.001
Jitter (local absolute) μs	204.67 (141.48 - 250.14)	134.99 (103.84 - 186.25)	621	0.003
Jitter (rap) %	1.7 (1.3-2.3)	1.1 (0.9-1.4)	478	< 0.001
Jitter (ppq5) %	1.8 (1.5-2.4)	1.2 (1.0-1.5)	468.5	< 0.001
Jitter (ddp) %	5.2 (4.1-7.0)	3.5 (2.8-4.4)	475.5	< 0.001
Shimmer (local) %	18.0 (16.5-19.8)	16.9 (14.7-17.7)	678	0.008
Shimmer (local, dB)	1.5 (1.5-1.6)	1.5 (1.4-1.5)	629.5	0.002
Shimmer (apq3) %	8.5 (7.5-10.0)	7.6 (6.3-8.5)	668	0.007
Shimmer (apq5) %	12.3 (11.1-13.6)	11.0 (9.8-12.5)	700	0.016
Shimmer (apq11) %	20.5 (18.1-22.1)	18.7 (16.3-22.3)	NS	0.185
Shimmer (dda) %	25.5 (22.5-30.0)	22.9 (19.1-25.7)	686	0.001
Autocorrelation (mean)	0.8 (0.74 - 0.82)	0.8 (0.79 - 0.85)	NS	0.103
Noise-to-harmonics ratio dB	0.3 (0.2-0.3)	0.2 (0.2-0.3)	NS	0.052
Harmonics-to-noise ratio dB	7.4 (5.6-8.4)	7.5 (7.0-0.5)	NS	0.196

Additionally, patients with laryngeal pathology were subclassified into four groups: malignant pathology (n = 9), benign pathology (n = 20), vocal cord paralysis (n = 12), and laryngopharyngeal reflux (n = 10). The Kruskal-Wallis test for independent samples was used to compare the groups, with Dunn’s correction applied to assess individual differences between groups when a statistically significant comparison was found. When evaluating the sustained vowel/a/, the mean CPPS in the malignant pathology, benign pathology, vocal cord paralysis, and laryngopharyngeal reflux groups were 5.58, 5.97, 5.69, and 8.42, respectively, with no significant differences found between subgroups (p = 0.142). Statistically significant differences were found in local jitter (average absolute difference between consecutive periods, divided by the average period) among the malignant neoplasms and laryngopharyngeal reflux groups, 0.66% vs. 4.02%, respectively (p = 0.009), and jitter rap (average absolute difference between a period and the average of this and its two neighboring periods, divided by the average period) amongst the malignant neoplasms and laryngopharyngeal reflux groups, 0.32% vs. 1.88%, respectively (p = 0.035).

When evaluating connected speech, the mean CPPS in the malignant pathology, benign pathology, vocal cord paralysis, and laryngopharyngeal reflux groups were 4, 7.65, 7.75, and 5.82, respectively, with no significant differences found among subgroups (p = 0.065). Statistically significant differences were found in jitter rap among the laryngopharyngeal reflux group (1.41%) and malignant neoplasms (2.19%), benign pathology (1.75%), and vocal cord paralysis (2.17%) groups (p = 0.035). 

## Discussion

The acoustic analysis is an attractive diagnostic method that can be routinely applied in voice examinations as it is reproducible, non-invasive, cost-effective, and objective. It allows for the assessment of voice quality, tone, and volume and enables comparison of results after treatment interventions [17,18]. The variables studied in conventional acoustic analysis are based on the periodicity of the captured signal, making it non-applicable in severely dysphonic patients. Some authors do not recommend performing connected speech analysis due to the presence of prosodic variations and muted phonemes inherent to the language. In our study, we found statistically significant differences in shimmer (18% vs. 16.9%, p = 0.008) and jitter (204.67 μs vs. 134.99 μs, p = 0.003) values among dysphonic and non-dysphonic patients using this type of voice sampling. Despite analyzing time-based parameters, we recommend conventional acoustic analysis as a complement to cepstral acoustic analysis in the routine evaluation of dysphonic patients.

CPPS acquisition has the advantage of not depending on the variations in the signal between vocal cycles, allowing for the assessment of vocal quality in connected speech and in severely dysphonic voices with greater reliability in a single parameter. A lower CPPS value is directly proportional to the severity of the dysphonia [19].

The heterogeneity in normative CPPS values exists due to variations among different languages, populations, sex, methodology, voice signals, and the performed software (with higher values obtained using Praat compared to ADSV) [20]. Buckley et al. reported normative values in an American population using Praat for men (sustained vowel/a/11.72 dB, sustained vowel/i/12.01 dB, continuous speech 6.40 dB) and women (sustained vowel/a/11.05 dB, sustained vowel/i/10.37 dB, continuous speech 6.49 dB), finding higher average values in men [15]. Sujitha et al., using the “Speech Tool” software, obtained similar results in Indian adults aged 30-40, reporting reference values for men (sustained vowel/a/7.73 dB, sustained vowel/i/6.28 dB, continuous speech 4.93 dB) and women (sustained vowel/a/7.63 dB, sustained vowel/i/5.55 dB, continuous speech 6.07 dB) [21]. In a Spanish-speaking population, Nuñez Batalla et al. also reported a significant difference in the average normative CPPS value for the sustained vowel/a/ among men and women of 16.4 dB, SD 2.8, and 11.0 dB, SD 2.22, respectively, with average ranges between 7.8 - 10.9 dB and 7.9 - 11.3 dB, respectively (sensitivity 70%, specificity 85% for dysphonia detection) [22]. Our study employed the Praat software, reporting in a non-dysphonic population a median CPPS of 11.39 dB (IQR 9.26-12.81) for sustained vowel and 6.38 dB (IQR 0.90-7.09) for continuous speech, finding lower values compared to those reported by Nuñez et al. We hypothesize several factors may influence our study results, such as anatomical differences in the vocal tract among ethnic groups, environmental factors (exposition to dialects and other languages), and phonologic intrinsic characteristics of language (as intonation or articulation of sound).

Significantly lower CPPS values have been reported in comparisons between dysphonic and non-dysphonic patients in various populations. Hasavand et al. reported significant differences in CPPS values between dysphonic and normophonic male subjects (sustained vowel/a/26.2 dB vs. 99.4 dB/continuous speech 25.86 dB vs. 100.32 dB) and female subjects (sustained vowel/a/25.5 dB vs. 100.5 dB/continuous speech 5.4 dB vs 2.5 dB) in the Iranian population using the "Speech Tool" software, attributing the lower values in the female population to the more frequent presence of a posterior glottic gap [23]. In Spanish adults, the use of Praat reported mean CPPS values for dysphonic subjects and controls in the sustained vowel/a/ of 11.52 dB vs 14.9 dB (p < 0.001) and 6.2 dB vs 7.9 dB (p < 0.001) in continuous speech, respectively [24]. There are reports in this population indicating pathological threshold values for dysphonic voice detection of 23.62 dB for the sustained vowel (S 87.5%, E 75.6%) and 18.4 dB for continuous speech (S 82.1%, E 86.6%) [25]. These results contrast with the values obtained in our population, despite speaking the same language, with a median of 6.59 dB (IQR 4.09-9.38) in sustained vowel phonation and 4.82 dB (IQR 3.57-6.03) in continuous speech for dysphonic patients. Regarding reports in the Latin population, our results are similar to those obtained by Rivera et al. in Colombian adults, finding mean CPPS values in continuous speech of 8.54 dB (SD 0.8975) for non-dysphonic patients and 6.4 dB (SD 0.9273) for dysphonic patients, respectively [26]. We believe such results were obtained as we share similar linguistic structure and racial demographic group characteristics, but further research among the Latin American population is needed to get normative threshold values of CPPS. 

This is the first comparative study of the cepstral analysis of the Mexican population with pathologic and non-pathological voice. Some limitations should be noted: this study was conducted at a single center with a relatively small sample and with a significant age difference between study groups. Additionally, the methodology did not include a perceptual auditory analysis to compare with the acoustic analysis and correlate with the severity of dysphonia in the study group.

## Conclusions

This study describes and compares for the first time the results of a vocal cepstral analysis in a Mexican adult population with laryngeal pathology against a control group. A low value of CPPS is a predictor of laryngeal pathology; therefore, its routinary use can be applied as a screening or diagnostic approach for a patient with vocal pathology. Further research is needed with a larger sample and a more uniform methodology to establish normative CPPS values in our population.
